# High-throughput adaptive sampling for whole-slide histopathology image analysis (HASHI) via convolutional neural networks: Application to invasive breast cancer detection

**DOI:** 10.1371/journal.pone.0196828

**Published:** 2018-05-24

**Authors:** Angel Cruz-Roa, Hannah Gilmore, Ajay Basavanhally, Michael Feldman, Shridar Ganesan, Natalie Shih, John Tomaszewski, Anant Madabhushi, Fabio González

**Affiliations:** 1 School of Engineering, Universidad de los Llanos, Villavicencio, Meta, Colombia; 2 Dept. of Computing Systems and Industrial Engineering, Universidad Nacional de Colombia, Bogotá, Cundinamarca, Colombia; 3 University Hospitals Case Medical Center, Cleveland, OH, United States of America; 4 Inspirata Inc., Tampa, FL, United States of America; 5 Hospital of the University of Pennsylvania, Philadelphia, PA, United States of America; 6 Cancer Institute of New Jersey, New Brunswick, NJ, United States of America; 7 University at Buffalo, The State University of New York, Buffalo, NY, United States of America; 8 Case Western Reserve University, Cleveland, OH, United States of America; Beijing University of Technology, CHINA

## Abstract

Precise detection of invasive cancer on whole-slide images (WSI) is a critical first step in digital pathology tasks of diagnosis and grading. Convolutional neural network (CNN) is the most popular representation learning method for computer vision tasks, which have been successfully applied in digital pathology, including tumor and mitosis detection. However, CNNs are typically only tenable with relatively small image sizes (200 × 200 pixels). Only recently, Fully convolutional networks (FCN) are able to deal with larger image sizes (500 × 500 pixels) for semantic segmentation. Hence, the direct application of CNNs to WSI is not computationally feasible because for a WSI, a CNN would require billions or trillions of parameters. To alleviate this issue, this paper presents a novel method, High-throughput Adaptive Sampling for whole-slide Histopathology Image analysis (HASHI), which involves: i) a new efficient adaptive sampling method based on probability gradient and quasi-Monte Carlo sampling, and, ii) a powerful representation learning classifier based on CNNs. We applied HASHI to automated detection of invasive breast cancer on WSI. HASHI was trained and validated using three different data cohorts involving near 500 cases and then independently tested on 195 studies from The Cancer Genome Atlas. The results show that (1) the adaptive sampling method is an effective strategy to deal with WSI without compromising prediction accuracy by obtaining comparative results of a dense sampling (∼6 million of samples in 24 hours) with far fewer samples (∼2,000 samples in 1 minute), and (2) on an independent test dataset, HASHI is effective and robust to data from multiple sites, scanners, and platforms, achieving an average Dice coefficient of 76%.

## 1 Introduction

The advent of whole-slide digital scanners has allowed for rapid digitization of histopathology slides, making these digitized slides images easy to store, visualize, share and analyze using computational tools. This rapidly growing field of Digital Pathology [1–3] is resulting in one of the newest forms of “big data”. Whole-slide images (WSI) in histopathology are large, typically each WSI could have a full spatial resolution of 80,000 × 80,000 pixels and approximately 20 GB in storage size at 40× magnification. Additionally, projects like the The Cancer Genome Atlas (TCGA) [4] have resulted in the creation of very large digital slide repositories. The TCGA currently hosts 11,079 cancer studies involving 34 different types of cancer and hosting over 1,095 Terabytes (∼1 Petabyte) of data [4]. This high volume of data requires the development and application of high throughput computational image analysis approaches for mining the digital image data. In particular, representation learning and deep learning approaches are the current state-of-the-art in several computer vision tasks such as object detection, object recognition and image annotation [5, 6]. Deep representation learning refers to a family of machine learning methods which attempt to learn multiple levels of representation to model complex relations among data. These methods attempt to discover more abstract features via higher levels of representation which then could help facilitate high-level decision tasks such as classification or prediction [5]. For image analysis, convolutional neural networks (CNN) is the most successful deep representation learning method. CNNs are multilayer neural networks, combining different types of layers (convolutional, pooling, classification) which then need to be trained in a supervised manner [5] for image analysis and classification tasks, which have focused on very small images [7–9].

Recently, fully convolutional networks (FCN) have shown the capability to extend CNN architectures, thereby achieving state of the art classification and segmentation performance for images of relatively small size [10, 11]. A fully convolutional network (FCN) is a neural network composed of convolutional layers without any fully-connected layer at the end of its network architecture. A convolutional neural network (CNN) is a neural network composed of convolutional layers and at least one fully-connected layer. FCNs can be seen as a generalization of CNNs. CNNs combine local information to make predictions at the global level. FCNs can make these predictions in a dense way at the pixel level. Each output pixel of a FCN can be seen as an individual CNN.

Some of the previous works have involved the application of CNN to histopathology image analysis [12–21] and very recently FCN was successfully applied to the problem of gland segmentation in colon histology images [22, 23]. However, these approaches have limited their analysis to small regions of interest (ROI) within the larger WSI. The main reason is that the overall size of the network depends on the size of the input image. For instance, a CNN with an input image of 200 × 200 pixels and 250 feature maps in the first convolutional layer would involve 10 million hidden units, while the same architecture with an input RGB color image of size 80,000 × 80,000 (a typical full resolution digitized WSI at 40× magnification) would require around 4.8 trillion hidden units, far exceeding the computational capabilities of most current high performance computing clusters by several orders of magnitude. Even a scaled down version (1:32) from the original full resolution WSI would require around 4.6 billions hidden units. This means that a direct application of the traditional CNN approach for object detection or pixel-level classification in WSIs for a full resolution or a scaled down version (1:32) is not tenable. The nearest alternative are FCNs as long as the image size can be allocated into the GPU memory [10, 11].

Some approaches have been proposed for tissue classification, tumor detection or grade scoring on WSIs [24–29]. However, most of the image processing tasks in WSIs for problems in digital pathology have focused mainly on image registration and preprocessing [30–32]. Increasingly, a number of deep learning approaches have begun to be applied for whole-slide histopathology image analysis [28, 29].

Precise invasive tumor delineation on the pathology slide is typically the first step for subsequent interrogation of tumor differentiation by the pathologist [33]. While approaches for breast cancer grading have been previously presented [34–36], these approaches require to define first the target ROI. Breast cancer (BCa) is the most common type of cancer in women and the second cause of death in developed countries [37]. Invasive BCa refers to those breast cancers that have spread from the original site and typically tend to have poorer prognosis [38].

This paper presents a High-throughput Adaptive Sampling for whole-slide Histopathology Image analysis (HASHI), a novel, accurate and high-throughput framework that combines the powerful capabilities of CNN models for image recognition and an adaptive sampling method for rapid detection of precise extent of invasive BCa on WSIs. The method is based on a CNN tile classifier which estimates the probability of the presence of invasive BCa within a WSI through adaptive sampling because CNN is only able to classify small regions, not the full WSI. Hence, instead of applying the tile classifier densely over the entire WSI, the method adaptively chooses regions with high uncertainty of a tissue tile being invasive or not. Regions of ambiguity tend to cluster on the border of the tumor regions, representing in most cases, a mixture of tumor and benign regions. The rationale behind HASHI is that regions where the predictor has a greater uncertainty about the type of tissue, will require more tile samples to be classified by the CNN in order to improve the confidence of the adaptive sampling method for those regions of ambiguity. Thus, homogenous regions tend to present the same morphological and architectural attributes within their local neighborhood and therefore low uncertainty about the type of tissue. While, heterogeneous regions tend to present mixtures of tissue types (invasive and non-invasive) with different morphological and architectural attributes within their local neighborhood representing high uncertainty about the type of tissue, and therefore requiring more tile samples. In this paper, we present a new sampling strategy that alternates between exploration and exploitation. The initial exploration involves a pseudorandom sampling in turn providing a coarse overview of the tissue type distribution in the WSI, distribution here representing the predictor likelihood associated with each tile. The regions identified as being ambiguous represent candidate regions for more dense local sampling or “exploitation”. This process is iterated several times. In this work we apply HASHI to the problem of automated detection and quantification of invasive BCa extent on WSIs. The HASHI classifier is trained with a cohort of nearly 500 patient studies drawn from multiple institutions and with a wide variation in staining and scanning attributes. The model is independently validated on a hold out test of almost 195 cases from the TCGA. Extensive results of model optimization and evaluation and parameter sensitivity are presented.

The rest of the paper is organized as follows: previous related works are described in Section 2; details of our approach are presented in Section 3; Section 4 details the experimental design; Section 5 presents the evaluation results and discussion; finally, in Section 6 we present our concluding remarks and directions for future work.

## 2 Previous related work

A number of recent histopathology image analysis methods have focused on identification of image features in conjunction with a machine learning classifier to predict presence or severity of disease from surgical or biopsy tissue specimens [3, 35, 36, 39–43]. Most approaches involving feature extraction from digital pathology images are based off a hand-crafted feature design. These hand-crafted features aim to capture different tissue morphologic and spatial properties including nuclear shape, nuclear architecture, color intensity, and tissue texture. Table 1 details a set of state-of-the-art hand-crafted features in histopathology image analysis and breast cancer digital pathology tasks [17, 36, 44]. These features are used as the baseline for comparative evaluation against HASHI approach based on CNN features.

**Table 1:** Set of hand-crafted features used for comparison against the CNN based feature learning approach.

ID	Category	Length	Features
CF	Color/intensity	56	First order statistics of 14 color channels [34, 35, 42].
GeF	Geometrical	48	First order statistics of geometrical / morphological features [35, 40, 42].
CH	Color Histograms	8 × 3	8-bin histogram for each RGB channel [35, 42].
SH	Shape Index Histogram	8 × 3	Shape index 8-bin histogram for each RGB channel [54].
MLBP	Multi-scale LBP	8 × 3	Multi-scale local binary patterns, 8-bin histogram for each RGB channel [42].
HF	Haralick features	26 × 3	First order statistics of 13 Haralick gray-level concurrence features from 4 orientations for each RGB channel [35, 42, 55].
RLF	Run-Length features	11 × 3	11 higher-order statistics of gray-level run-length matrices properties at 4 orientations for each RGB channel [40].
GWF	Gabor wavelet features	71 × 3	First order statistics of 71 Gabor filters from 8 orientations for each RGB channel [34, 35, 42, 55].
TGF	Topography / Graph features	51	Voronoi diagram (12), Delaunay triangulation graph (8), minimum spanning tree (4) and nuclei (27) [34, 40, 42, 45].

While work on hand-crafted feature design is an active area of research, these features tend to be sensitive to staining and scanning artifacts [3, 42, 45]. Hand-crafted features are approximations, based on mathematical and statistical formulations, of the visual content designed by human experts according to prior knowledge of the visual regions of interest. Consequently these features may not capture all the relevant characteristics and complex relationships embedded within the disease patterns manifest on histopathology images [46]. By contrast, representation learning aims to automatically learn the transformation of data that facilitates high-level prediction and classification tasks using one level or multiple levels of representation (i.e. deep learning) [5–7]. Compared to hand-crafted features, representation learning based approaches attempt to learn the most appropriate representation directly from the data. While these approaches tend to be domain agnostic (i.e. not specifically invoking visual features that represent the domain), they are focused on identifying image features geared towards maximizing high-level classification tasks in pattern recognition. More recently, fully convolutional networks (FCN), and other types of CNNs [10, 11, 47, 48], have been shown to outperform state-of-the-art approaches for semantic segmentation tasks involving natural images. FCN is an extension of CNN architectures for pixel wise prediction resulting in high-level salient maps for each class. However, these models are trained using the same CNN architecture as employed by patch-based learning approaches.

Recently, approaches based on representation learning and deep learning have been applied for histopathology image analysis, either in a supervised or unsupervised manner [13–16, 18–21, 29, 49–51]. Most previous studies have been based on supervised learning (e.g. tumor, mitosis and tubule nuclei detection [13–16, 20, 51]), with relatively few approaches being geared towards unsupervised learning [16, 49, 50, 52]. In fact, the most successful representation learning approaches in histopathology image analysis have been supervised approaches involving CNNs, outperforming hand-crafted features in several problems [53]. Recently, FCNs have been successfully applied to the problem of gland segmentation in colon histology images [23].

Kothari et al. [26] provided an excellent review of the state of the art in image analysis and classification tasks related to histopathological whole-slide imaging informatic methods. They describe how most approaches to feature analysis of WSIs are typically limited to manually selected ROIs. In [24], the authors describe a multi-resolution framework for tile-based tissue classification to determine the grade of neuroblastomas. Kothari et al. [25] proposed a visualization framework for studying visual morphological patterns across 1,301 histopathological WSIs from 571 patients with ovarian serous cystadenocarcinoma from TCGA. In [27], the authors assessed the impact of different classification algorithms and features sets on both accuracy and computing time for quantification of necrosis in WSIs. Huang et al. [28] attempted to address the problem of time-efficient determination of the nuclear pleomorphism score from breast cancer WSIs. They used a sparse coding approach for unsupervised learning of the visual representation of the content in the WSIs and then combined this representation with first- and second-order statistics of multivariate Gaussian distributions. These statistics were then employed in conjunction with a support vector machine classifier to identify invasive and non-invasive cancer patches over WSIs, albeit at a low magnification. ROIs are then selected from regions that secure a higher nuclear pleomorphism score using a dynamic sampling based on Voronoi tessellation. The final nuclear pleomorphism score is calculated from higher-scaled versions of the ROIs selected. Finally, we successfully applied CNN with a regular/dense sampling over WSI to predict invasive tumor regions of BCa but spending a lot of computing time [29].

The main limitations of these previous approaches has been that the analysis has been limited to small ROIs within the larger WSIs or performing time-consuming regular/dense sampling. Additionally, hand-crafted features tend to be very specific to particular domains or data sources and not seamlessly generalizable to different tasks or applications. Finally most of these approaches have involved evaluating the methods on a relatively small cohort of cases typically originating from a single institution. Consequently it is not clear whether these approaches will actually be useful for routine clinical practice. In contrast, our method has the following advantages and makes the following contributions: i) accurate and reproducible detection of invasive breast cancer regions on new unseen WSIs, ii) ability to generalize to images acquired from different data sources and domains, and iii) a new high-throughput adaptive sampling method that makes our approach feasible for WSIs and is an order of magnitude more efficient compared to a naive implementation of CNNs, while not compromising detection accuracy. In order to explicitly address the issues of variability in staining, slide preparation, and scanning across multiple sites, our training and validation sets were comprised of slide images from multiple different institutions.

## 3 Methodology

### 3.1 Brief overview of HASHI

**Fig 1:**
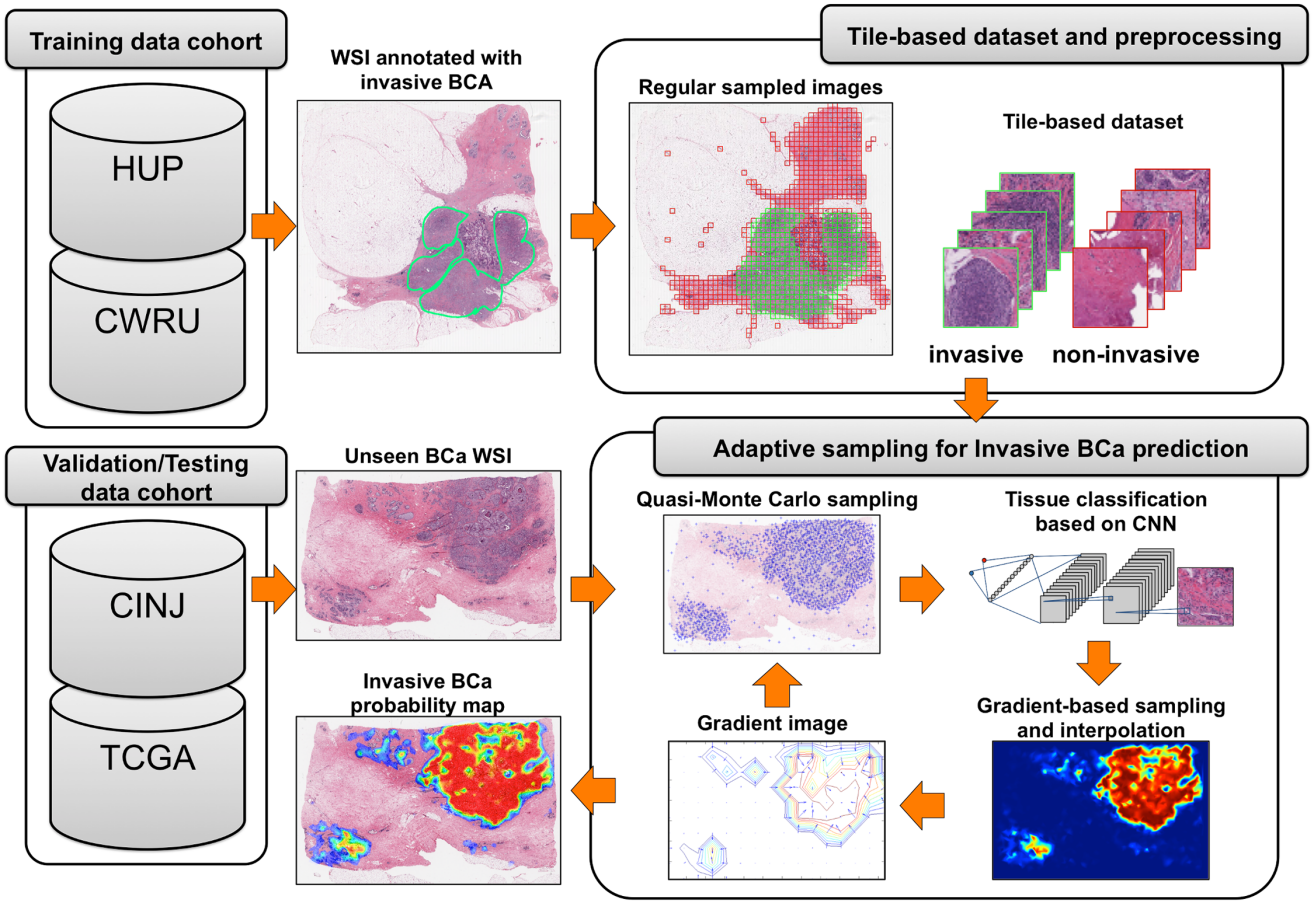
Overview of HASHI method. Overview of the high-throughput adaptive sampling for whole-slide histopathology images method (HASHI) based on CNNs for automated detection of invasive breast cancer (BCa) in WSIs. Training data cohorts: Hospital of the Univ. of Pennsylvania (HUP) and Case Western Reserve Univ. (CWRU). Validation/Testing data cohorts: Cancer Institute of New Jersey (CINJ) and The Cancer Genome Atlas (TCGA).

Fig 1 presents the general overview for the HASHI framework for invasive BCa detection in WSI. Training exemplars for the CNN are generated by pathologists on digitized WSIs. The training phase of the CNN uses as input, a tile-based dataset obtained by applying a regular sampling of WSIs from the training data cohort. This process is used to extract tiles of a fixed square size both from pathologist annotated invasive and non-invasive tissue regions. The prediction stage on new unseen WSIs involves the following steps: first, tiles are extracted from the WSI using pseudorandom sampling; the CNN classifier is applied to each tile; the prediction produced by the CNN is used to build an interpolated probability map which is then used to identify regions where the classifier has high uncertain with regard to the tissue type or class (invasive or not). These regions are then determined as needing a more dense sampling. This is achieved by choosing the high gradient magnitudes of the probability map associated with the tumor borders; the newly sampled exemplars are used to produce an improved probability map estimation; the process is iterated producing a final invasive BCa probability map. The details of each step are explained in the following subsections.

### 3.2 Adaptive gradient-based sampling

Algorithm describes the adaptive gradient-based sampling strategy, which iteratively refines an initial coarse estimation of an invasive BCa probability map. Inputs to the algorithm include a WSI *X*, the algorithm parameters: maximum iterations *T* and number of sample points per iteration *N*. The algorithm begins with a tile sampling process resulting in the generation of *N* tiles. Each tile is classified using the CNN-trained model *M* to obtain the probability of the presence of invasive BCa at the particular location occupied by each tile. By interpolating the probabilities calculated at each tile, a probability map *P* for the WSI is obtained. In order to determine regions with higher uncertainty, the gradient, *G*, of the probability map is calculated. *G* is then used to prioritize the sampling selection of new tiles for the next iteration. The process is repeated until the maximum number of iterations *T* is reached.

**Algorithm:** Adaptive gradient-based quasi-Monte Carlo sampling

**INPUT:**

 *M*: CNN-trained model

 *X*: WSI

 *T*: maximum iterations

 *N*: number of samples per iteration

 *samples* ← pseudorandom sampling (*X*, *N*)

 **for** *i* = 1 to *T* **do**

  *predictions* ← tile classification (*M*, *samples*)

  *P* ← invasive BCa probability map interpolation (*predictions*, *samples*)

  *G* ← probability gradient (*P*)

  *samples* ← gradient based sampling (*G*, *X*, *N*)

 **end for**

 **return** invasive BCa probability map *P*

### 3.3 Tile-based CNN classifier training

#### 3.3.1 Ethics statement

Data analysis was waived review and consent by the IRB board, as all data was being analyzed retrospectively, after de- identification. All experimental protocols were approved under the IRB protocol No. 02-13-42C with the University Hospitals of Cleveland Institutional Review Board, and all experiments were carried out in accordance with approved guidelines.

#### 3.3.2 Tile-based dataset construction and preprocessing

Similar to [29], a regular sampling was performed on each WSI from the training set to extract tissue samples for the training of the tile-based classifier. Only tiles corresponding to tissue regions were included, fatty tissue and slide background regions were ignored. The criterion for considering that a tile is non-tissue is based on the standard deviation and average statistics of the illumination of the tile. If the standard deviation is close to zero, i.e. homogenous color, and the average close to 255, i.e. white color, it is considered to be a non-tissue tile. Additionally a tile sample was considered to be a positive example (i.e. invasive BCa) if a certain pre-defined proportion of its area overlaps with the region manually annotated by pathologists as being invasive tumor, otherwise it is labeled as a negative example (i.e. no cancer) [15]. Each image patch or tile is converted from RGB to YUV color space and normalized to a mean of zero and variance of one. The color space transformation and normalization allow for decorrelation and accentuation of the differences between the input image tiles, independently of the color variability. This approach therefore helps accelerating the process of gradient-based learning during the training stage [5].

#### 3.3.3 Tile-based CNN classifier

**Fig 2:**
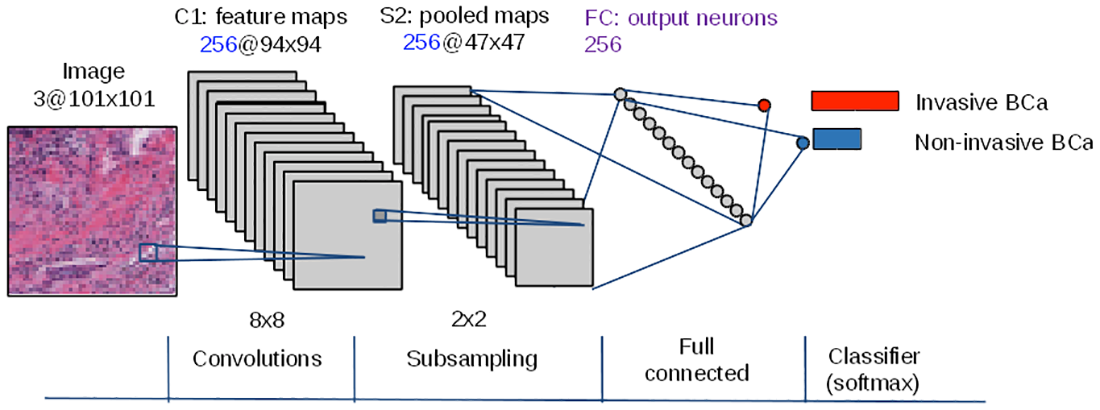
Illustration of the CNN architecture used to distinguish between invasive and non-invasive breast cancer (BCa) image tiles. The architecture is a 2-layer CNN with 256 neurons in the first layer convolutional (C1) and subsampling/pooling layer (S2) and 256 neurons in the fully-connected layer (FC), (i.e. CS256-FC256). Amongst the various architectures considered, this architecture was selected because it has a good trade-off between classification performance and a shallower architecture (fewer layers).

Using the same methodology from [29], we trained three different CNN architectures (this is detailed in Section 4.2), such as it was presented in [29], the best architecture identified was found to be a 2-layer CNN (CS256-FC256), illustrated in Fig 2. This architecture is composed of a convolutional and a pooling layer of 256 units followed by a fully-connected layer of 256 units. The classification layer is a softmax classifier with two outputs (invasive and non-invasive) activated by the softmax function. Since this is a two-class problem, softmax functions are therefore equivalent to logistic functions. The convolution layer involves application of a 2D convolution of the input image with a kernel of 8 × 8 pixels. The pooling (or subsampling) layer applies a spatial L2-pooling function without overlapping, employing a pooling kernel of 2 × 2 pixels for each feature map obtained from the convolution step. An advantage of the L2-pooling function is that it allows the learning of local translationally invariant features [56, 57]. The output of the pooling layer is fed to a fully-connected layer followed by a final classification layer. The training process uses the set of tiles sampled from both the invasive and non-invasive tissue regions. The CNN model is then trained using a stochastic gradient descent approach [5] in order to minimize a softmax loss function (Eq 1):

$$\begin{array}{c} L\left(W\right)=-\frac{1}{m}\left[\sum_{i=1}^{m}\sum_{j=1}^{C}1\left\{y^{\left(i\right)}=c\right\}\log\frac{e^{w_{c}s^{\left(i\right)}}}{\sum_{l=1}^{C}e^{w_{l}s^{\left(i\right)}}}\right]+\frac{\lambda }{2}{∥W∥}_{F}^{2}, \end{array}$$ (1)

where *m* is the number of training examples, *C* is the number of classes, $W\in R^{C\times n}$ are the weights of the network in the last layer with *w*_*c*_ as the vector associated to class *c*, *s*^(*i*)^ = *f*(*x*^(*i*)^) is the feature vector for example *i* and *x*^(*i*)^ is the output of the full-connected layer, $y^{\left(i\right)}\in N$ is the label associated to example *i* and *λ* is the regularization parameter. 1{*statement*} function outputs 1 if *statement* is true, 0 otherwise. The CNN training process involves searching for a weight vector *W* which aims to minimize the loss function (Eq 1). The implementation of the CNN model, its training and testing were performed using Torch 7, a scientific computing framework for machine learning [58].

### 3.4 Adaptive prediction of invasive BCa regions

In order to predict the likelihood of individual tiles representing invasive cancer, the classifier would need to be repeatedly applied to each tile in the WSI. For a WSI of size 80,000 × 80,000 pixels, a tile sampling approach involving patch sizes of 101 × 101 pixels translates to over 6.39 × 10^9^ predictions, which is clearly computationally infeasible. Hence our approach involves making predictions on a sample of patches from the WSI and then extrapolating from these predictions to the whole image. Traditional ways of performing this sampling include: dense, regular and random with a uniform distribution [59]. The method presented in this paper uses an adaptive scheme which performs a guided sampling that focuses on those image areas with higher uncertainty. Each of the individual steps involved in the adaptive tile based classification are described below.

#### 3.4.1 Tile sampling

The goal of this step is to select a set of tiles (each of 101 × 101 pixels) from the WSI, which will be used to create a probability map over the entire WSI. The tiles may be selected by deterministic (dense or regular) or random sampling. The different sampling approaches are discussed here below.

**Regular sampling** This strategy involves sampling tiles at equally spaced intervals on a regular grid. For instance, given a WSI of *K* × *K* size and tiles of *k* × *k* size, the step size *s*, in both the X and Y directions is 1 ≤ *s* ≤ *k*. The extreme case involves using a step size *s* = 1, which means an expected number of samples of (*K* − *k*)^2^. This case corresponds to a dense sampling of the WSI.

**Uniform random sampling** Regular sampling is deterministic. An obvious alternative strategy is random sampling, i.e. to select the tiles using random coordinates generated from a particular probability distribution. Without *a priori* knowledge of the image content, a uniform probability distribution is a natural choice for the random sampling algorithm.

**Quasi Monte Carlo sampling** Uniform random sampling may involve over-sampling tiles in some regions of the image while leaving other regions under-represented. This may not be the most efficient strategy since the predictions on overrepresented regions tend to be redundant. Quasi Monte Carlo (QMC) sampling represents a good compromise between regular and random sampling. QMC sampling enables an efficient sampling strategy and a regular spatial exploration. The random sampling procedure employs deterministic (pseudo-random) sequences designed to have low discrepancy, where discrepancy is a measure of the uniformity of a distribution of finite point sets [60]. This property is an advantage for QMC in contrast to Monte Carlo methods (based on random sampling) since QMC does not result in clumping (i.e. accumulation of samples in a small area), which in turns results in better accuracy for the sampling process [60]. We chose the Sobol and Halton sequences [61] for our iterative adaptive sampling method. With these sequences it is possible to incrementally add sample points without discarding those already previously generated.

#### 3.4.2 Invasive BCa probability map estimation

The sampled tiles are fed to the CNN classifier to determine the probability of the presence of invasive or non-invasive BCa in each particular tile. Cubic interpolation is then applied to extend this estimation to all the pixels in the WSI, resulting in an invasive BCa probability map at the end of each iteration.

#### 3.4.3 Probability gradient

A gradient image ∇*P* is calculated to identify the directional changes of the probability map *P* as follows:

$$\begin{array}{c} \nabla P=\frac{\partial P}{\partial x}\hat{x}+\frac{\partial P}{\partial y}\hat{y} \end{array}$$ (2)

where $\frac{\partial P}{\partial x}$ is the gradient in the *X* direction and $\frac{\partial P}{\partial y}$ is the gradient in the *Y* direction. Then, the gradient magnitude image $\left|\nabla P\right|=\sqrt{{\left(\frac{\partial P}{\partial x}\right)}^{2}+{\left(\frac{\partial P}{\partial y}\right)}^{2}}$ is calculated to identify regions with high or low variations among tissue types in the probability map *P*. High values correspond to a heterogeneous spatial distribution of both invasive and non-invasive tissue types along tumor boundaries (strong changes), whereas low values correspond to a homogeneous distribution of either invasive or non-invasive tissue types (soft changes). Thus, the magnitude |∇*P*| has low values if the local spatial regions of *P* have similar values. In contrast, the magnitude |∇*P*| is high, if the local spatial regions of *P* dramatically change their probability values.

#### 3.4.4 Gradient-based sampling selection

The probability gradient enables a more intelligent spatial sampling of points, with a more dense sampling directed at high magnitude regions within the gradient map (representing transitional areas from one tissue type to another), while fewer samples are extracted from homogeneous regions (possibly representing a single tissue type region). This is accomplished by the following procedure: first, 2*N* samples are generated using either the random or QMC sampling mechanism; second, the samples are ranked according to the magnitude of the gradient; finally, the top *N* samples are returned.

## 4 Experimental design

### 4.1 Breast cancer data cohorts

The data used in this study are H&E-stained histological slides from patients with estrogen receptor-positive (ER+) breast cancer. The images correspond to slides from four different sites. The WSIs were digitized with Aperio or Ventana scanners. We used only those images which had been scanned in at a 40x magnification (i.e. 0.2456 *μm* per pixel for Aperio and 0.23 *μm* per pixel for Ventana). Images were further downsampled by a factor of 32:1. As illustrated in Table 2, different data cohorts were used for training, validation or testing. The training and validation sets were used for model parameter tuning and optimization. Independent model evaluation was performed on the hold out test set.

**Table 2:** Breast cancer data cohorts used for training, validation and testing in the experimental evaluation.

ID	Site	Cases	Scanner	Dataset
*D*_1_	Hospital of the Univ. of Pennsylvania	239	Aperio	Training
*D*_2_	Univ. Hospitals Case Medical Center/Case Western Reserve Univ.	110	Ventana	Training
*D*_3_ (*D*_1_ + *D*_2_)	Hospital of the Univ. of Pennsylvania and Univ. Hospitals Case Medical Center/Case Western Reserve Univ.	349	Aperio, Ventana	Training
*D*_4_	Cancer Institute of New Jersey	40	Aperio	Validation
*D*_5_	Cancer Institute of New Jersey (subset)	12	Aperio	Validation
*D*_*test*_	The Cancer Genome Atlas (https://tcga-data.nci.nih.gov/)	195	Aperio	Testing

Three expert pathologists (NS + MF, HG) independently provided the ground truth annotations of invasive breast cancer regions on digitized WSIs for each data cohort (NS + MF for *D*_1_, *D*_4_ and *D*_5_; HG for *D*_2_ and *D*_*test*_; NS + MF and HG for *D*_3_). The pathologists manually delineated the invasive regions at 2x magnification using the viewing software ImageScope v11.2 from Aperio and Image Viewer v3.1.4 from Ventana.

### 4.2 Experiment 1: Comparing CNN vs handcrafted features

Using the same methodology from [29], the goal in this experiment was to compare the most commonly used hand-crafted features in histopathology image analysis for breast cancer diagnosis, (Table 1) [35, 40, 42, 44, 62], against different CNN based architectures for tile-based tissue classification of invasive BCa. This experiment uses as training data set the *D*_3_ data cohort (349 cases) and *D*_4_ as test data cohort (40 cases). Parameter tuning was performed using cross validation over the *D*_3_ training dataset. The performance of the classifier was evaluated using the area under the receiver operating characteristic curve (AUC). Both training and testing were performed using a NVIDIA^®^GPU Tesla^™^ C2050 (448 Cores, 2.6 GB Memory).

Each hand-crafted feature listed in Table 1 was combined with each of two classifiers: random forests (RF) and support vector machines (SVM). For RF, the training step involved optimizing the parameters corresponding to the number of trees, while for the SVM, different kernel functions were evaluated: linear, radial basis function (RBF), intersection, Chi-square (*χ*^2^), and Jenson-Shannon’s. For the CNN-based approach, three different architectures were evaluated, such as in [29]. The first was the architecture employed in [15] which was a 3-layer CNN, called ConvNet. This architecture comprises of 16 neurons in the initial convolutional and pooling layers, 32 neurons in the second stage, and 128 neurons in the third fully-connected layer (CS16-CS32-FC128). The second architecture explored was the one that was previously successfully applied to the problem of mitosis detection in breast cancer histopathology images [13], which comprises four layers of convolutional and pooling neurons with 16 neurons in each, and a fully-connected layer of 128 neurons (CS16-CS16-CS16-CS16-FC128). The third architecture explored was a 2-layer CNN with 256 neurons in the first layer and 256 neurons in the fully-connected layer (CS256-FC256) (Fig 2).

In order to determine the statistical significance of the difference in performance between methods, we applied a multiple comparison Kruskal-Wallis test using the following procedure: we built 100 different data groups with 60% of the instances randomly chosen from *D*_3_, applying bootstrap sampling. The AUC for each method trained from each of 100 different training datasets was evaluated on *D*_4_. Then, the methods were ranked according to their performance for each data group. Based on the rankings, the Kruskal-Wallis test statistic was calculated and a post-hoc Tukey’s honestly significant difference criterion was applied to check for pairwise differences between methods.

### 4.3 Experiment 2: Evaluating the impact of the sampling strategy on the effectivity and efficiency of probability map prediction

Seven different sampling methods were evaluated to determine the more efficient strategy in terms of both detection accuracy and computing time. The baseline sampling method chosen was (a) the regular sampling (*regular*) which takes equally-spaced samples by varying the step size. For the random and pseudo-random sampling methods, we evaluated (b) uniform random sampling (*uniform*), (c) quasi-Monte-Carlo sampling using the Sobol sequence (*qmc-sobol*), and (d) quasi-Monte-Carlo sampling using the Halton sequence (*qmc-halton*). In addition, sampling strategies which involve using image gradients to identify regions of uncertainty, were combined with the previous sampling strategies, (e) gradient-based uniform sampling (*grad-uniform*) and (f) gradient-based quasi-Monte-Carlo sampling, using either Sobol (*grad-qmc-sobol)* and (g) Halton (*grad-qmc-halton*) sequences. All sampling approaches (with and without incorporation of gradient image information) were applied iteratively with the same set of parameters: 20 iterations and 100 samples per iteration, resulting in 2000 samples for each sampling approach. This experiment used the best performing CNN model identified in Experiment 1 in conjunction with all the various sampling strategies that were evaluated. The performance of HASHI for each sampling strategy was evaluated on *D*_5_. The classification of the tiles with the CNN model was done using only a single CPU core in a Intel 64-bit Linux server (12 CPU cores, 28GB). This was done in order to simulate the type of general purpose computing environment that one might expect to see in a typical pathology clinical practice, one that does not avail of any special purpose high end hardware.

The output of our method is an invasive BCa probability map over the WSI, i.e. a measure of the probability of presence of invasive BCa for each pixel in the WSI. The probability map is used to calculate a predicted region with invasive BCa, by selecting those pixels where the probability is above a given threshold.

Additionally, an equivalent FCN architecture based on the best CNN model obtained from Experiment 1 was evaluated to evaluate FCN with respect to HASHI for generating the invasive cancer probability map.

Each scheme was quantitatively evaluated by measuring the Dice coefficient [63] between the predicted BCa region and the ground truth annotation from the expert pathologist. The Dice coefficient is defined as follows: $Dice=\frac{2|P\cap G|}{\left|P|+|G\right|}$, where *P* corresponds to the predicted region by our method, and *G* is the ground truth binary mask obtained via the pathologists annotation. For each of seven sampling strategies, the average Dice coefficient over the test data set is calculated for a different number of samples.

### 4.4 Experiment 3: Evaluating performance on a hold-out independent test set

We trained a CNN model with the best configuration found in Experiment 1. Additionally a linear SVM was also trained with the best performing handcrafted features (CF). Both approaches were trained with *D*_3_ and evaluated on *D*_*test*_. The evaluation on *D*_*test*_ was performed using a NVIDIA^®^GPU Tesla^™^ C2050 (448 Cores, 2.6 GB Memory).

The performance measures used to evaluate and compare the different methods were Dice coefficient (Dice), positive predictive value (PPV), negative predictive value (NPV), true positive rate (TPR), true negative rate (TNR), false positive rate (FPR), and false negative rate (FNR).

## 5 Results and discussion

### 5.1 Experiment 1: Comparing CNN vs handcrafted features

Table 3 shows the AUC values (mean and standard deviation) for the best performing three CNN models described in Subsection 4.2 (*CNN*_1_, *CNN*_2_, *CNN*_3_) evaluated on *D*_4_. Also shown in Table 3 are the best performing models of hand-crafted features (see Table 1) combined with RF and SVM classifiers (*M*_1_ to *M*_7_). The experimental results in Table 3 show that the three CNN classifiers outperform the best combinations of hand-crafted features and machine learning classifiers. Additionally, the CNN classifiers exhibit a smaller variance in terms of the AUC measure compared to hand-crafted features.

**Table 3:** Comparison between CNN models and state-of-the-art hand-crafted features trained with *D*_3_ and evaluated on *D*_4_ in terms of AUC.

ID	Methodology	AUC
*CNN*_1_	CS16-CS16-CS16-CS16-FC128	**0.9021 ± 0.0097**
*CNN*_2_	CS256-FC256	**0.9018 ± 0.0093**
*CNN*_3_	CS16-CS32-FC128	**0.8915 ± 0.0093**
*M*_1_	CF + SVM-Linear	0.8711 ± 0.0947
*M*_2_	RLF + SVM-Linear	0.8689 ± 0.0963
*M*_3_	CH + SVM-Linear	0.8448 ± 0.1047
*M*_4_	SH + SVM-Linear	0.8444 ± 0.1065
*M*_5_	HF + SVM-Linear	0.8385 ± 0.0942
*M*_6_	TGF + SVM-Linear	0.7998 ± 0.1068
*M*_7_	RLF + RF	0.7985 ± 0.0892

The multicomparison Kruskal-Wallis test, using a post-hoc Tukey’s honest significant difference criterion, reveals that there is no statistical difference (*p* < 0.05) in terms of critical difference among the CNN classifiers (*CNN*_1_, *CNN*_2_, *CNN*_3_). Mean differences above the critical difference are suppose to be statistically significant. Additionally the two top performing CNN models significantly outperformed the best performing hand-crafted features. Such as in [29], the subsequent experiments and evaluation we employed *CNN*_2_ (i.e. CS256-FC256), since it has a simpler architecture (fewer layers).

### 5.2 Experiment 2: Evaluating the impact of the sampling strategy on the effectivity and efficiency of probability map prediction

**Fig 3:**
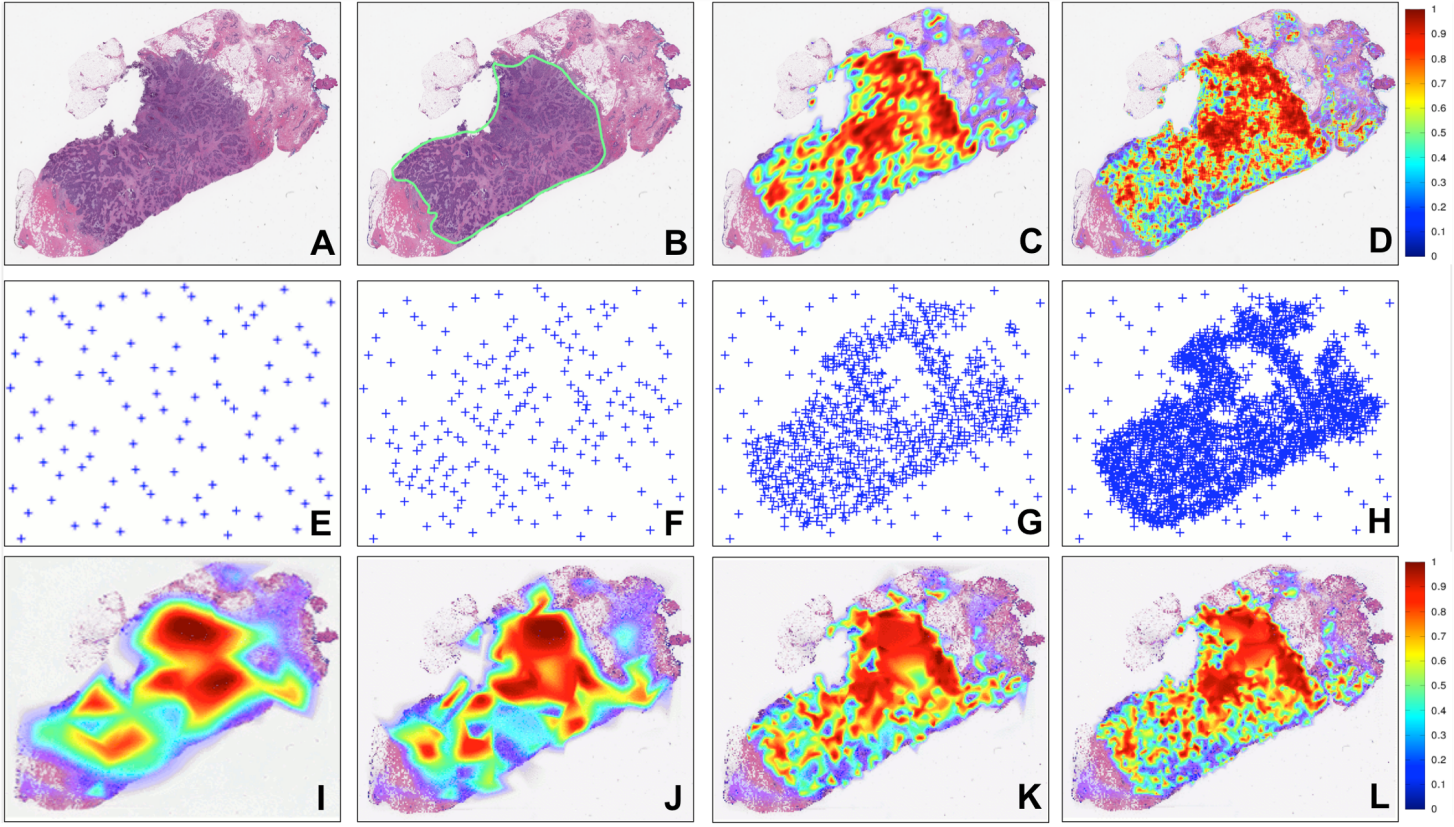
Comparison between sampling methods (*regular* and *dense*) with HASHI using gradient-based quasi-Monte Carlo sampling (*grad-qmc-halton*) [59, 61]. The new unseen WSI (A) with its corresponding ground truth annotation from an expert pathologist (B). The probability maps using regular sampling with a step size equal to the patch size (C) and regular dense sampling with step size equal to 1 pixel (D). HASHI involves an iterative process of extracting patch samples (E-H) and obtaining the corresponding probability maps (I-L) for the 1st (E, I), 2nd (F, J), 8th (G, K) and 20th iteration respectively (H, L).

**Fig 4:**
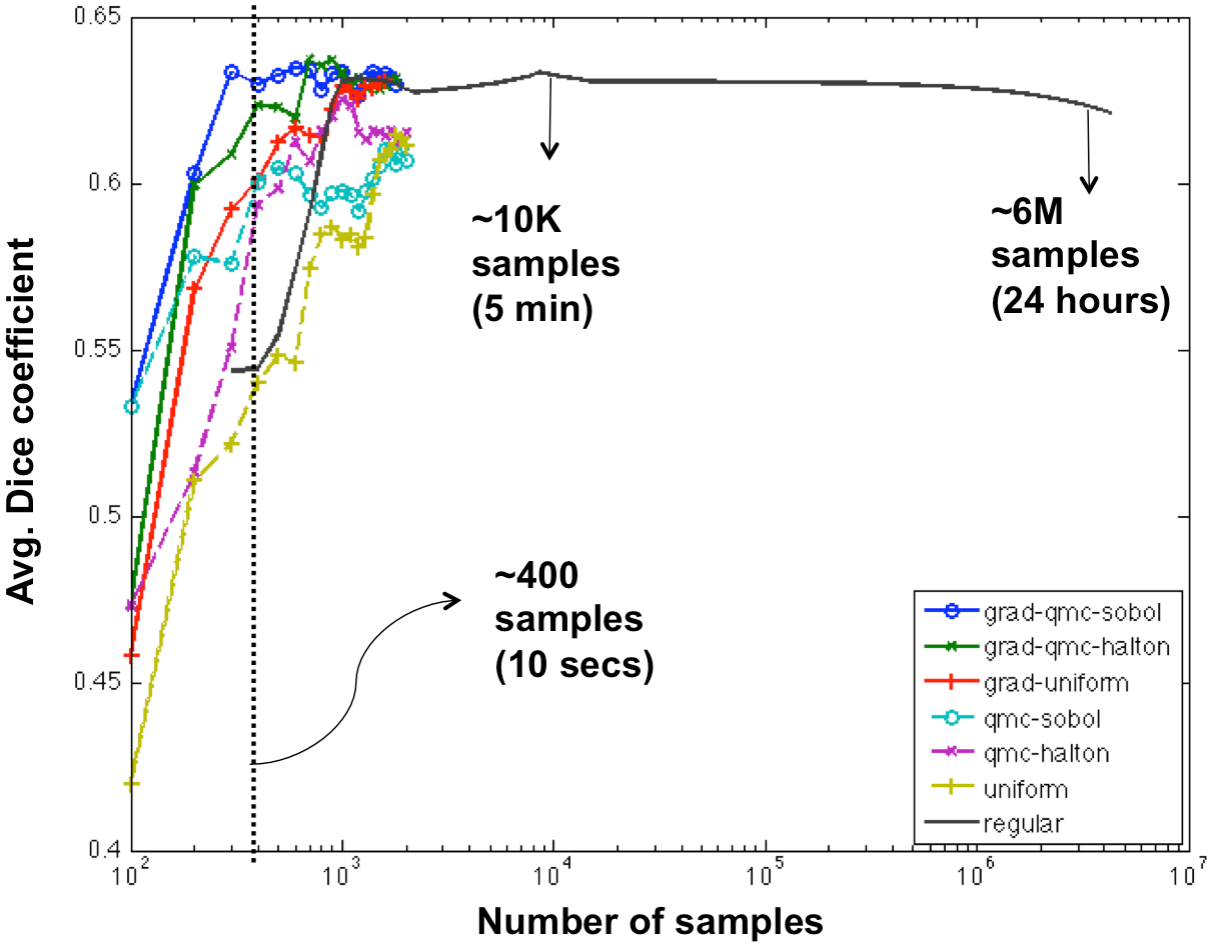
Quantitative evaluation of the different sampling strategies in terms of average Dice coefficient (y-axis) versus the number of samples (x-axis) used. All strategies were trained with *D*_3_ and evaluated with *D*_5_.

Fig 3 shows the invasive BCa probability map produced for a representative WSI using the sampling strategies presented in Subsection 4.3. Fig 3A shows a test WSI while Fig 3B shows the ground truth annotation provided by an expert pathologist. Fig 3C is the prediction of the invasive BCa probability map using *CNN*_2_ and regular grid sampling with a step size of 50 pixels. While this sampling strategy is fast (31 secs), the resulting probability maps are extremely coarse and imprecise. Fig 3D shows the invasive BCa probability map obtained using *CNN*_2_ and dense regular sampling, which is the extreme case of regular grid sampling where the step size is 1 pixel. The resulting probability map is highly specific and detailed, but unfortunately with a run time of 22 hours it is also quite unfeasible for application in a clinical setting. Fig 3E-3L show the iterative process using *CNN*_2_ and the new adaptive sampling method (*grad-qmc-halton*). Fig 3E-3H illustrate the sampled points for the *CNN*_2_ classification process at iterations 1, 2, 8 and 20, respectively. As may be appreciated from Fig 3I-3L, *grad-qmc-halton* sampling yields a result that appears comparable to that obtained via dense sampling. This is also reflected in the quantitative evaluation results shown in Fig 4.

Fig 4 shows the quantitative results obtained for the different sampling strategies in order to predict the extent and location of invasive BCa regions as a function of the number of tile samples required per iteration. The cases from *D*_3_ were used to train *CNN*_2_ and those from *D*_5_ were used to evaluate the probability map prediction corresponding to *CNN*_2_ and to each of the different sampling strategies. The x-axis, in a logarithmic scale, corresponds to the number of tile samples required by each sampling method and the y-axis corresponds to the Dice coefficient. Note that the number of samples (x-axis) is proportional to the computing time. Each sampling strategy is depicted as a line. The regular sampling strategy was evaluated using different step sizes (200, 150, 100, 75, 50, 25, 1 pixels), while the random and pseudo-random sampling strategies (*uniform*, *qmc-sobol*, *qmc-halton*) were evaluated using a total of 20 iterations, with 100 samples employed per iteration.

The experimental results reveal that adaptive sampling (*grad-qmc-sobol*, *grad-qmc-halton* and *grad-uniform*) not only outperforms regular sampling and non-adaptive random sampling (*uniform*, *qmc-sobol*, *qmc-halton*), but also manages to achieve the same detection performance as dense sampling but with a substantial reduction in the overall computation time. While dense sampling employs an average of 6 million tile samples, with a corresponding compute time of around 24 hours per WSI, our adaptive sampling strategies (*grad-qmc-sobol* and *grad-qmc-halton*) achieve a comparable detection performance while only employing 2000 samples and a corresponding run time of less than one minute per WSI using only CPU for *CNN*_2_ predictions.

**Fig 5:**
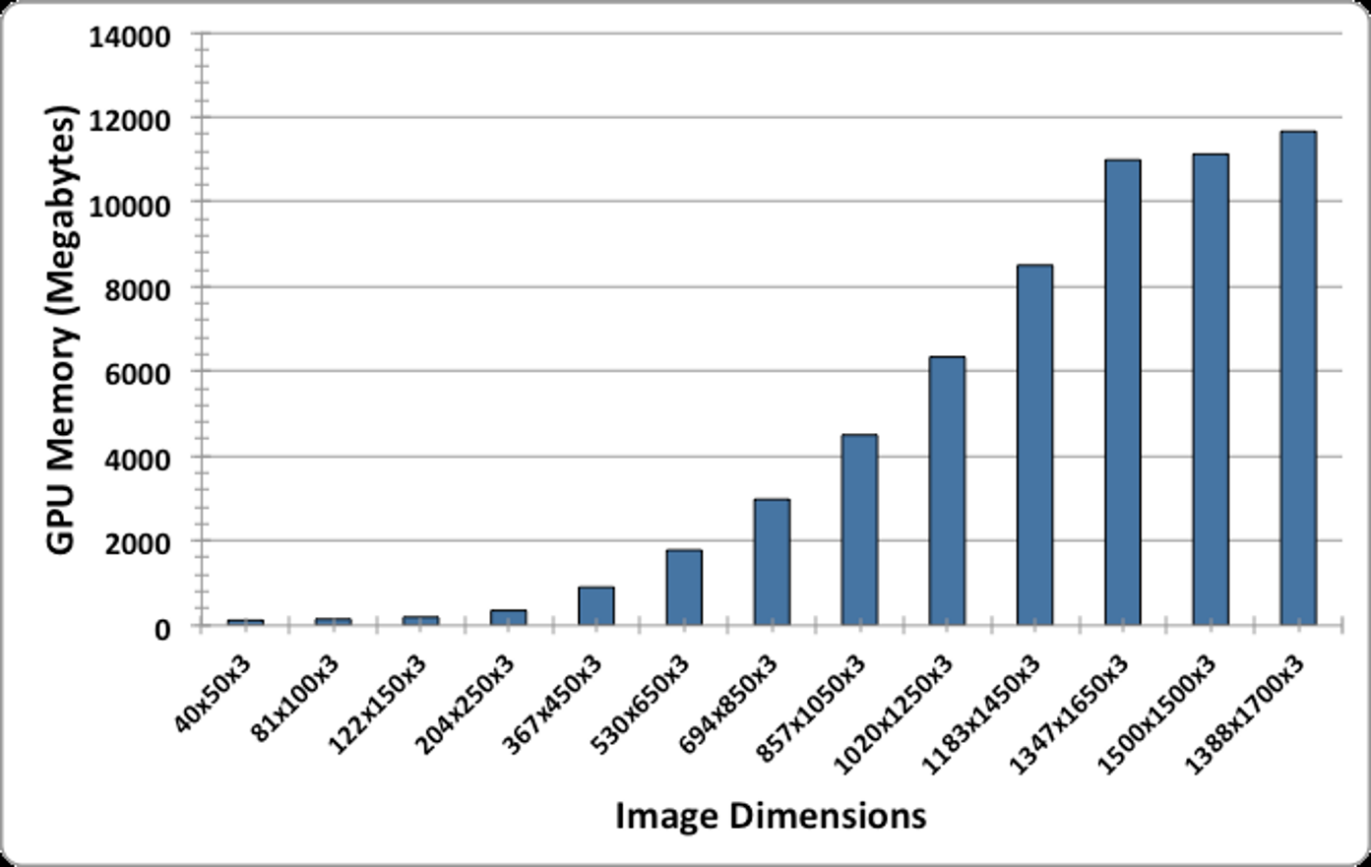
GPU memory size requirements (Megabytes) for different image dimensions (height × width × channels) for the experimentation of FCN based on *CNN*_2_ model.

Additionally, HASHI was compared against the equivalent architecture of *CNN*_2_, one employed by a FCN. Fig 5 shows the comparison between image dimensions and the GPU memory requirements for prediction. This experimentation was performed on a NVIDIA^®^ GPU Titan X with a GPU memory of 12GB because it allows to allocate larger image sizes for invasive probability maps generation in contrast to the GPU used for training that only support image sizes of 530 × 650 pixels.

This reveals that the generation of the invasive probability map is limited by the GPU memory size. The smaller image size in *D*_5_ had 2431 × 1853 pixels, which is a larger image size than the image sizes that can be allocated in GPU memory according to the experiments of Fig 5. Hence, in order to compare the performance of our approach against FCN, the test images had to scaled down to make it possible to invoke the FCN approach. Thus, the generation of the probability map for the larger image analyzed by the FCN (i.e. ∼1500 × 1500) took 1 second.

Table 4 shows the invasive BCa detection performance comparison between HASHI and FCN. We report the average and standard deviation of the Dice coefficient values for both approaches for images within *D*_5_. The parameter configuration for HASHI involved 20 iterations with 100 samples per iteration using the *CNN*_2_ model. For FCN, the equivalent architecture based on *CNN*_2_ model was used, but the images had to be scaled because of the previously described constraint with the GPU memory. While the mean performance for FCN is 4% higher compared to HASHI, the difference between the two approaches was not found to be statistically significantly different across all images in *D*_5_.

**Table 4:** Invasive BCa detection performance of *HASHI* and the equivalent FCN architecture on *D*_5_ in terms of Dice coefficient.

	Dice
*HASHI*	0.67 ± 0.22
*FCN*	0.71 ± 0.21

### 5.3 Experiment 3: Evaluating performance on a hold-out independent test set

We applied the best combination of tile-based tissue classifier (CS256-FC256) and adaptive sampling method *(grad-qmc-halton*). HASHI was subsequently evaluated on *D*_*test*_. Table 5 summarizes and compares the performance of *HASHI* versus the best classifier obtained with the hand-crafted features (i.e. *M*_1_) using the same adaptive sampling method *(grad-qmc-halton*), in terms of average Dice, TPR, TNR, FPR and FNR. A more detailed analysis of the distribution of Dice coefficient per case reveals that for most of WSI, HASHI had a Dice coefficient between 0.7 and 0.9 and an overall median value of 0.8228, whereas the best classifier based on hand-crafted features achieved a median value of 0.8007. Some of the cases with the lowest Dice coefficient were because the classifier also identified ductal carcinoma in situ (DCIS), a stage 0 breast cancer that is considered as a pre-malignancy. However, since we set a very stringent requirement on only identifying invasive cancer, the detection of DCIS was deemed to be a false positive error. Most other cases with a low Dice coefficient corresponded to slides with a poor quality of staining.

**Table 5:** Invasive BCa detection performance of our method on the *D*_*test*_ testing dataset in terms of Dice, PPV, NPV, TPR, TNR, FPR, FNR.

	Dice	PPV	NPV	TPR	TNR	FPR	FNR
*HASHI*	**0.76 ± 0.20**	**0.72 ± 0.22**	**0.97 ± 0.05**	**0.87 ± 0.16**	**0.92 ± 0.08**	**0.08 ± 0.08**	**0.13 ± 0.16**
*M*_1_ + *grad*-*qmc*-*halton*	0.73 ± 0.21	0.68 ± 0.24	0.96 ± 0.05	0.86 ± 0.19	0.91 ± 0.08	0.09 ± 0.08	0.14 ± 0.19

**Fig 6:**
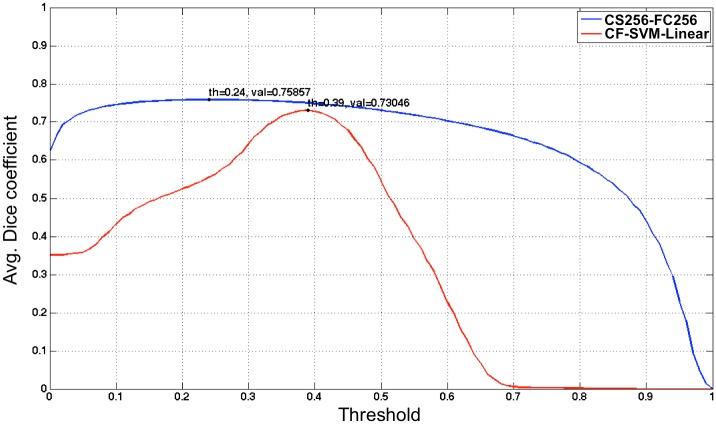
Performance comparison between *HASHI* and *M*_1_ in terms of Dice coefficient in the independent *D*_*test*_ test data cohort by varying the classification threshold of the invasive BCa probability map.

**Fig 7:**
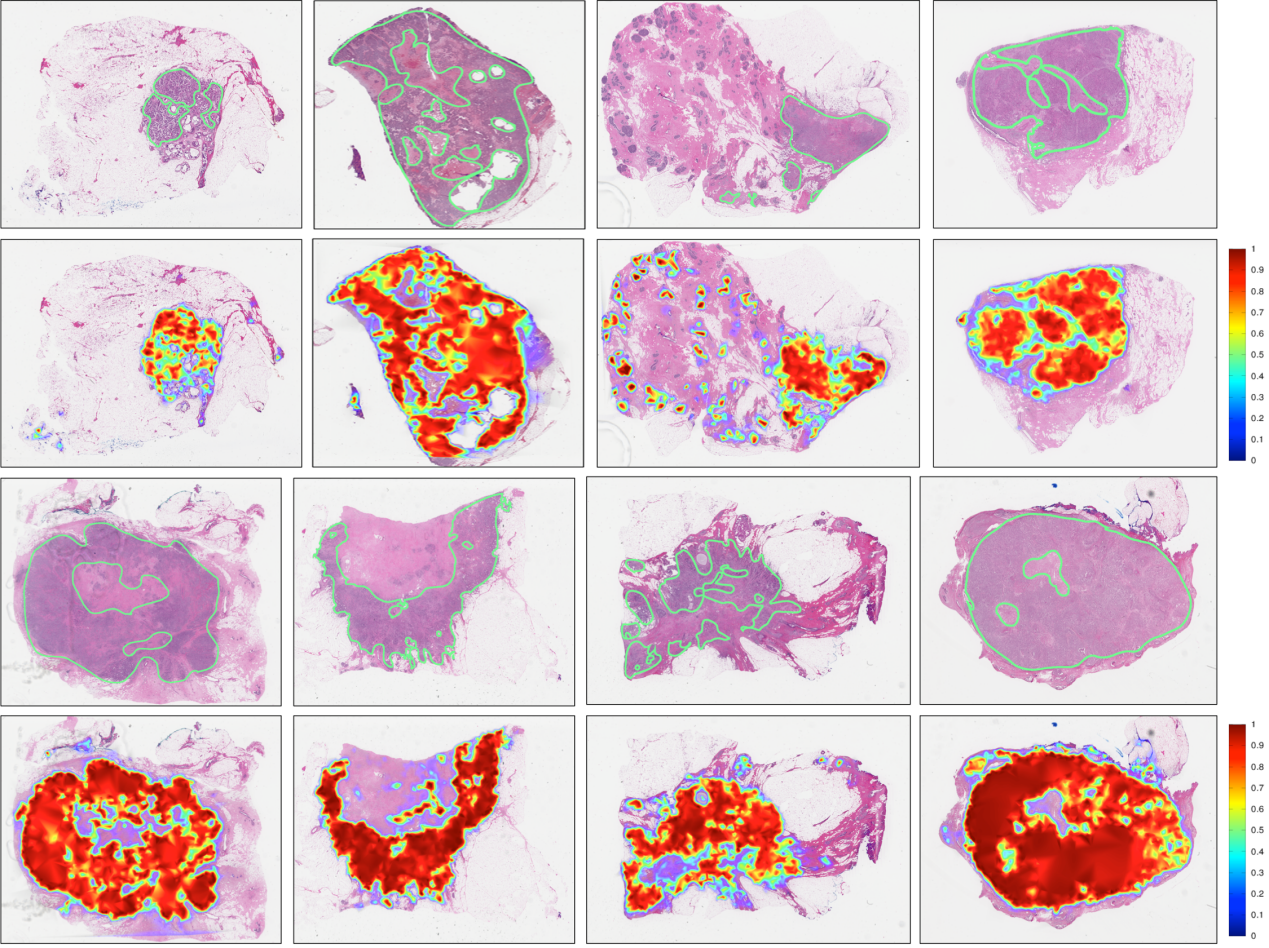
Results of the invasive BCa probability maps (second and fourth rows) predicted by *HASHI* on representative WSIs from *D*_*test*_ compared to the ground truth annotations from expert pathologists (first and third rows). Red regions represent locations identified by HASHI as having a high likelihood of cancer presence while the blue regions represent the lowest likelihood of cancer presence.

Fig 6 shows the detection sensitivity of the threshold value from classifier of both *HASHI* and *M*_1_ with *grad-qmc-halton* as evaluated on *D*_*test*_. Interestingly, the performance of *HASHI* is more stable and robust, achieving a greater than 0.7 Dice coefficient, for most of the threshold values employed and achieving an optimal average Dice coefficient of 0.7586 at a threshold of 0.24. By contrast, *M*_1_ in conjunction with *grad-qmc-halton* was found to be more sensitive to the thresholds, achieving good results only in the interval between 0.35 and 0.45 with a best result of 0.7305 at a threshold of 0.39. Fig 7 shows good concordance between predictions of HASHI and pathologists annotations of invasive cancer (ground truth) for representative slides chosen from *D*_*test*_.

## 6 Conclusions

This paper presented a novel accurate and high-throughput method (HASHI) for automatic invasive breast cancer detection in WSIs. While several previous works have proposed to use deep learning methods for histopathology image analysis, these approaches tend to be computationally expensive. Additionally these approaches typically do not deal with WSIs and only involve analysis of small ROIs. In contrast, our new approach HASHI is able to employ state-of-the-art CNNs models to classify tissue regions through an efficient and smart new adaptive sampling method. We addressed the challenges of high complexity and visual variability of tissues, invasive and non-invasive, and the large size of WSIs by combining a state-of-the-art image analysis technique, CNNs, with an efficient adaptive sampling strategy. The model was trained to learn the most appropriate representation using nearly 600 WSIs from 4 different institutions. To deal with large-size images, we developed a novel adaptive sampling method which integrates quasi-Monte-Carlo sampling with a gradient-based adaptive strategy which focuses sampling on those areas with higher uncertainty.

The method was systematically evaluated using nearly 200 interdependent validation studies from the TCGA. The results revealed that our approach is effective and robust, with reproducible results across data from different sources. The experimental results also ratified that CNN models outperform hand-crafted state-of-the-art feature analysis approaches, several of which have been recently employed for different tasks in histopathology image analysis. In addition, our new adaptive sampling method was shown to yield comparable detection accuracy while having a computationally efficiency that was more than 3 orders of magnitude (>1500×) faster compared to dense sampling. In addition, an evaluation of new fully convolutional networks (FCN) [10, 11] was performed to compare against HASHI. FCN was found to be more computationally efficient and yielded a marginally higher, but not statistically significant detection accuracy. Unfortunately one of the caveats of the FCN approach is that it can only be run on smaller images (no larger than 1500 × 1500 pixels). This is a concern with whole slide images which can typically have sizes above 50*K* × 50*K* pixels. Additionally higher resolution images are typically required for several tasks in breast cancer pathology, such as grading, tubule and mitosis counting to name a few [13, 14, 17, 34–36]. In contrast, HASHI does not have special hardware requirements, HASHI can be applied without a GPU card and could take advantage of commodity CPU hardware to do the processing. Additional speed-up can be achieved by using multi-core processing, potentially making this an approach that could be more conducive for a clinical pathology workspace.

We do however acknowledge that the work had some limitations. Firstly the approach was unable to distinguish DCIS from invasive BCa. While DCIS is considered as zero stage of breast cancer, it is not invasive even though it is sometimes considered as a pre-malignancy. However, since we set a very stringent requirement on only identifying invasive cancer, the detection of DCIS was deemed to be a false positive error. The other few cases with low prediction performance of invasive BCa were primarily in slides with poor staining quality.

The approach presented in this paper has potential to serve as a decision support tool to help pathologists to speed up breast cancer identification and localization, significantly alleviating their workload.

Future directions include, extending our dataset to involve manual ROI annotations of DCIS and other tumor confounding non-malignant presentations. Also, we will seek to potentially combine our approach with an FCN approach in conjunction with GPUs to further speed up the analysis and interrogation of large whole slide images.

## Supporting information

S1 Fig Animation of *HASHI* iterative process of extracting patch samples and obtaining the corresponding probability maps. (GIF)

S2 Fig High magnification WSI example from TCGA with the ground truth annotation from expert pathologists. (TIFF)

S3 Fig High magnification WSI example from TCGA with the invasive probability map prediction obtained by *HASHI*. (TIFF)
