# Supplementary figures and images for: High-throughput adaptive sampling for whole-slide histopathology image analysis (HASHI) via convolutional neural networks: Application to invasive breast cancer detection
Source: PLoS One. 2018 May 24;13(5):e0196828. doi: 10.1371/journal.pone.0196828 (PMC5967747; doi:10.1371/journal.pone.0196828)

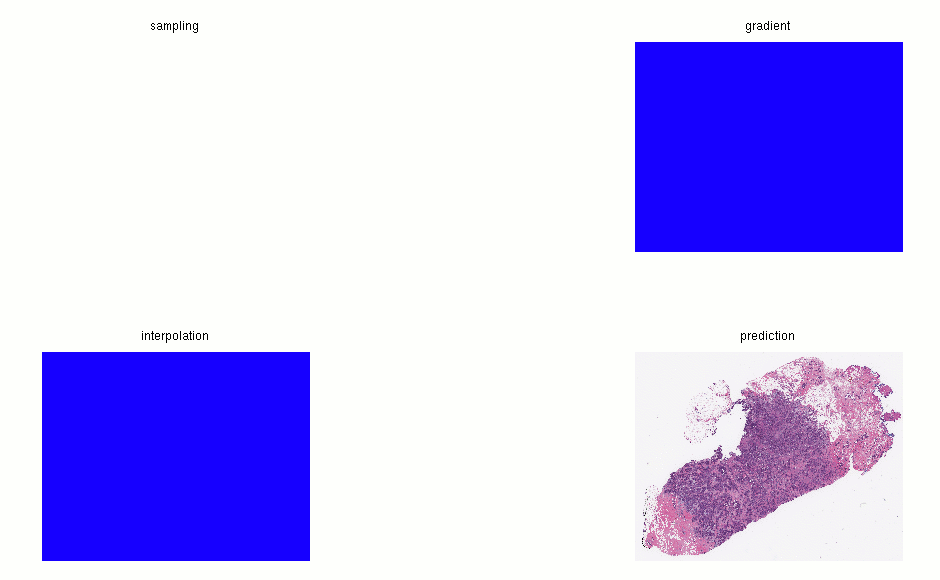

Supplement: S1 Fig — (GIF) [file pone.0196828.s001.gif]

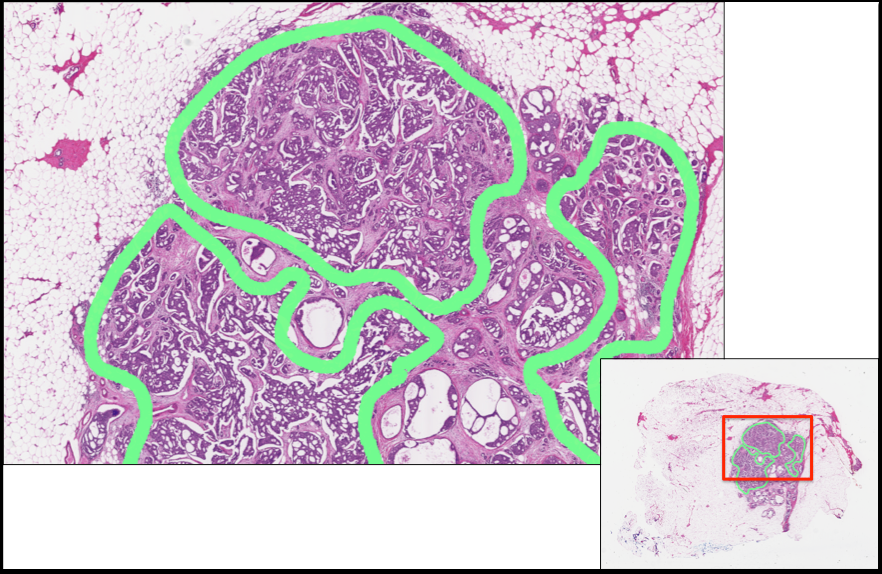

Supplement: S2 Fig — (TIFF) [file pone.0196828.s002.tiff]

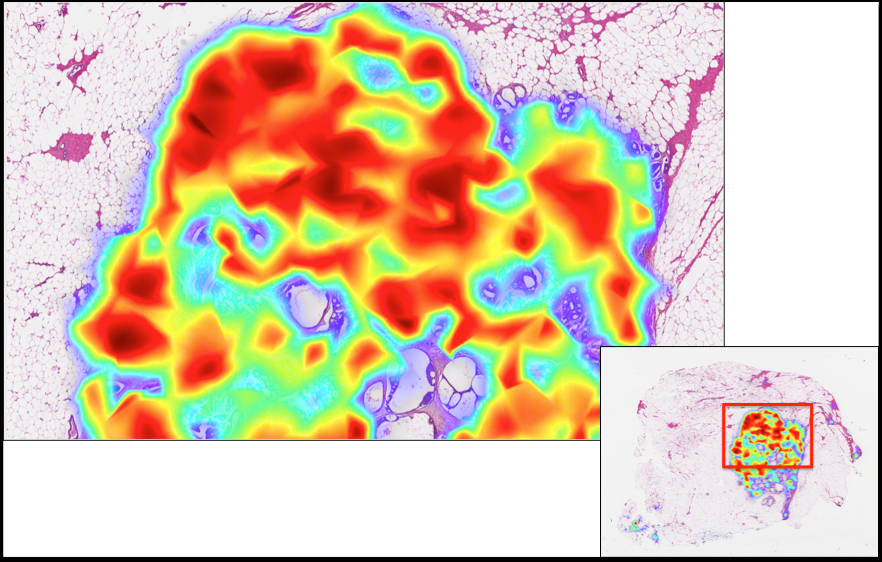

Supplement: S3 Fig — (TIFF) [file pone.0196828.s003.tiff]
